# A Web-Based Toolkit to Provide Evidence-Based Resources About Crystal Methamphetamine for the Australian Community: Collaborative Development of Cracks in the Ice

**DOI:** 10.2196/mental.8891

**Published:** 2018-03-20

**Authors:** Katrina Elizabeth Champion, Cath Chapman, Nicola Clare Newton, Mary-Ellen Brierley, Lexine Stapinski, Frances Kay-Lambkin, Jack Nagle, Maree Teesson

**Affiliations:** ^1^ National Health and Medical Research Council Centre of Research Excellence in Mental Health and Substance Use National Drug and Alcohol Research Centre University of New South Wales Sydney Australia; ^2^ Department of Preventive Medicine Feinberg School of Medicine Northwestern University Chicago, IL United States; ^3^ Priority Research Centre for Brain and Mental Health The University of Newcastle Callaghan Australia; ^4^ The Real Drug Talk Melbourne Australia

**Keywords:** methamphetamine, substance-related disorders, internet, preventive psychiatry, health education

## Abstract

**Background:**

The use of crystal methamphetamine (ice) and the associated harms for individuals, families, and communities across Australia has been the subject of growing concern in recent years. The provision of easily accessible, evidence-based, and up-to-date information and resources about crystal methamphetamine for the community is a critical component of an effective public health response.

**Objective:**

This paper aims to describe the codevelopment process of the Web-based *Cracks in the Ice Community Toolkit*, which was developed to improve access to evidence-based information and resources about crystal methamphetamine for the Australian community.

**Methods:**

Development of the *Cracks in the Ice Community Toolkit* was conducted in collaboration with community members across Australia and with experts working in the addiction field. The iterative process involved the following: (1) consultation with end users, including community members, crystal methamphetamine users, families and friends of someone using crystal methamphetamine, health professionals, and teachers (n=451) via a cross-sectional Web-based survey to understand information needs; (2) content and Web development; and (3) user testing of a beta version of the Web-based toolkit among end users (n=41) and experts (n=10) to evaluate the toolkit’s acceptability, relevance, and appeal.

**Results:**

Initial end user consultation indicated that the most commonly endorsed reasons for visiting a website about crystal methamphetamine were “to get information for myself” (185/451, 41.0%) and “to find out how to help a friend or a family member” (136/451, 30.2%). Community consultation also revealed the need for simple information about crystal methamphetamine, including what it is, its effects, and when and where to seek help or support. Feedback on a beta version of the toolkit was positive in terms of content, readability, layout, look, and feel. Commonly identified areas for improvement related to increasing the level of engagement and personal connection, improving the ease of navigation, and balancing a “low prevalence of use, yet high impact” message. A total of 9138 users visited the website in the 3 months immediately post launch, and over 25,000 hard-copy *Cracks in the Ice* booklets and flyers were distributed across Australia. Of these resources, 60.93% (15,525/25,480) were distributed to relevant organizations and mailing list subscribers, and 39.07% (9955/25,480) were ordered directly by individuals, services, and community groups via the *Cracks in the Ice* website.

**Conclusions:**

The codevelopment process resulted in an engaging Web-based resource for the Australian community to access up-to-date and evidence-based resources about crystal methamphetamine. The *Cracks in the Ice Community Toolkit* provides much-needed information and support for individuals, families, and communities.

## Introduction

### Background

The use of methamphetamine and related harms has been the subject of growing concern among the Australian community and media in recent years. Although methamphetamine is not a new drug in the Australian market [1,2], growth in the availability and consumption associated with the high-purity, crystalline form of methamphetamine (crystal methamphetamine or ice) has caused considerable concern among the community. Recent data suggest that there has been a shift toward using crystal (ice) rather than powder (speed) forms of methamphetamine among regular methamphetamine users [3,4] as well as an increase in methamphetamine-related harms [2] and deaths [5]. Indeed the physical, psychological, and social harms associated with methamphetamine use are significant. Major harms include increased risk of stroke and other cardiovascular problems, dependence, psychosis and other mental health problems, violence, and overdose [5-9].

In April 2015, the Australian Government established a National Ice Taskforce to provide advice to the government on the impacts of crystal methamphetamine in Australia and the actions needed to address this growing problem. Findings from the taskforce indicated that there was a clear need to support families, workers, and communities to better respond to drug and alcohol issues, including crystal methamphetamine use [10]. One critical aspect of responding to this issue is the provision of easily accessible, evidence-based, and up-to-date information and resources about crystal methamphetamine for the general population. Over the past 10 to 15 years, there has been a rapid growth in the number of individuals with access to the internet, with almost 40% of the world’s population now having access to online information [11]. In 2017, 86% of Australia’s population were internet users [12] and an estimated 80% of Australians use the internet to search for health information [13]. The potential of the internet to improve accessibility and to overcome geographic and physical constraints makes it a medium of growing importance for the dissemination of health information, support, and treatment [14], including in relation to substance use and mental health problems [15]. With similar rates of internet access reported among active users of illicit drugs [16], the potential for technology to reach marginalized groups in the community is large.

### Objectives of This Study

Although there has been a proliferation of information about crystal methamphetamine in Australia, both in the media and on the Web, the credibility of such information is not always apparent, and it is likely difficult for members of the community to evaluate what is evidence-based and what is not. Thus, as part of a coordinated response to the National Ice Taskforce recommendation, the *Cracks in the Ice Community Toolkit* [17] was developed. The Web-based toolkit was designed to improve access to evidence-based and up-to-date information and resources about crystal methamphetamine for Australians, including concerned community members, users, their friends and family members, health professionals and emergency service workers, and schools. This paper summarizes the comprehensive codevelopment and beta-testing process of the *Cracks in the Ice Community Toolkit.*

## Methods

### Overview of the Codevelopment Process

Development of the *Cracks in the Ice Community Toolkit* was a broad-reaching and iterative process, involving multiple phases over an 18-month period. As an initial step, an Expert Advisory Group (EAG), consisting of researchers with expertise in drug and alcohol prevention and treatment, internet interventions, and intervention development, was established to provide guidance and advice throughout the development process. Collaborations with consumer experts (ie, individuals with a lived experience of addiction, especially related to ice use, or mental health issues) were also established to provide advice throughout the development process. For example, consumer experts contributed to initial brainstorming, provided feedback on preliminary designs and different iterations of the Web-based toolkit, reviewed the beta website, and provided ideas for future development. Consumer participation is important to ensure that consumer needs, concerns, and values are not overlooked [18,19]. Engaging and linking with consumers was a critical component of the codevelopment process and ensured a strong end user voice in the final *Cracks in the Ice* toolkit.

The development process was informed by the latest available literature and data on crystal methamphetamine use [2,4,7,8,20-22] and drug education [23] and was developed in collaboration with community members across Australia and expert collaborators. Development occurred across 3 specific phases, which are outlined below:

1. Initial consultation with end users (Australian general population, including concerned community members, people who use crystal methamphetamine, their families and friends, health professionals, and teachers) via a Web-based survey
2. Content and Web development, in consultation with the EAG and consumer experts
3. Beta testing among end users and the EAG to ascertain feedback about the acceptability and relevance of the *Cracks in the Ice Community Toolkit*.

**Figure 1:**
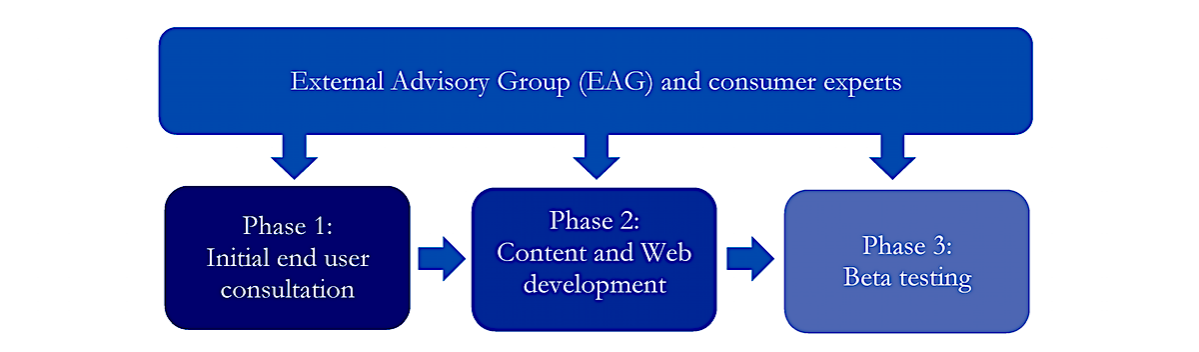
The codevelopment process of the *Cracks in the Ice* Community Toolkit.

The codevelopment process is outlined in Figure 1, and key phases are summarized in further detail below.

### Phase 1: Initial End User Consultation

#### Design and Procedure

An anonymous Web-based self-report survey was conducted with members of the Australian community between December 2015 and January 2016. Recruitment occurred via paid Facebook advertisements over a 2-week period that invited participants to *“* help to develop a website to improve access to information and support about methamphetamine. *”* Eligible participants were individuals aged 16 years or older who currently resided in Australia, and all participants were required to provide informed consent before completing the survey. The survey sought to better understand information needs about crystal methamphetamine among the Australian community, and to gain feedback about preliminary design concepts and preferences to inform development of the Web-based toolkit. At the end of the questionnaire, all participants were given the opportunity to provide their email address to enter the draw to win an Apple iPad; email addresses were not linked to survey response data. All aspects of the study were approved by the UNSW Sydney Human Research Ethics Committee (HREC; HC15732). A full copy of the questionnaire is available on request.

#### Measures

Demographic data collected included gender, age, place of residence, occupation, and country of birth. Lifetime use of methamphetamine was assessed using 2 items: *“* Have you ever used methamphetamine (also known as ice, speed, base, crystal meth, meth, shabu, Tiny, goey or glass)? *” (Yes or No)* and *“* What type of methamphetamine have you used *?” (Ice [crystal], speed or base).* Respondents who reported crystal methamphetamine use were asked a further set of items to assess route of administration (*smoking, injecting, other*), history and frequency of use, reasons for using, and intentions to use in the future. Harms associated with crystal methamphetamine use were assessed among the whole sample using an adapted version of the Social Harm from Others Scale [24]. These items were assessed to inform development of content for the Web-based toolkit, particularly for family and friends of people who use crystal methamphetamine. Participants were also asked to indicate reasons for visiting an information website about crystal methamphetamine, information areas of most interest, and preferences for how and where to seek help related to ice use. Finally, all respondents were asked to provide feedback on preliminary design concepts (eg, logos, webpage layout, and designs) to be included in the Web-based toolkit.

#### Data Analysis

Data were collated and analyzed in IBM SPSS Statistics 22 (IBM Corp, Armonk, NY, USA). Descriptive analyses were conducted to illustrate the sample characteristics and summarize interest in components of the proposed toolkit.

### Phase 2: Content and Web Development

Content development occurred at multiple stages throughout the development process. First, a preliminary site structure was developed in consultation with the EAG to identify key content areas and target audiences for the Web-based toolkit. Feedback on the structure was collected during Phase 1, with results from end users confirming the key areas and target groups proposed by the EAG. A key aim of this project was to bring together the best available evidence and resources about crystal methamphetamine rather than creating entirely new material. As such, the next step in the content development process was to review existing resources developed by members of the project team for the *Positive Choices* portal [23,25]. This portal houses evidence-based drug education resources and factsheets for school students, parents, and teachers, including those related to methamphetamine, which were developed in relation to the latest available literature. These factsheets were used as the basis for the development of the *Cracks in the Ice* key webpages, with adaptations made as necessary (eg, adapting content to reflect a general population rather than a school-based audience). Scholarly databases and relevant websites were also searched using keywords related to methamphetamine (eg, “crystal methamphetamine,” “ice,” “ice information”) to identify other relevant resources to inform development of webpage content. All new content was developed by a member of the project team with reference to existing evidence-based resources and the relevant research literature and were subject to expert review by at least two members of the EAG.

#### Assessment of Eligibility of External Resources

Existing resources developed by individuals or groups external to the research team, such as guidelines and Web-based programs, were independently reviewed by the *Cracks in the Ice* project team. Resources were assessed for eligibility for inclusion using an adapted version of the National Health and Medical Research Centre Body of Evidence Matrix [26] against 4 specific criteria: (1) evidence base, (2) impact and utility, (3) generalizability, and (4) applicability (see Multimedia Appendix 1 for additional information). In addition, resources were only eligible for inclusion if they were less than 10 years old at the time of development (ie, developed during 2006-2016), unless a justification could be made as to why the resource should be included (eg, no other existing resources were available or the resource addresses an important area of need). To avoid duplication of content, resources were ineligible if their content substantially overlapped with other resources already included on the *Cracks in the Ice* website.

#### Readability Testing

To assess whether the vocabulary to be used in the developed content was appropriate, potentially problematic words, identified by 2 members of the research team, were checked for their frequency of use in the English language, as an indication of how well known and understood they would be by a general population audience. This was conducted using the Oxford English Dictionary Key to Frequency tool, where word frequency is ranked between Band 1 (extremely rare) and Band 8 (very common). All words within Bands 5 and 8 were permitted for inclusion on the website. Words in Bands 1 to 3 were excluded or replaced. Band 4 words, described by the Oxford English Dictionary as “recognisable to English speakers,” were assessed on a case-by-case basis and included if they were deemed to be the most appropriate term to use. Terms that described specific mental health or physical health conditions, for example, were included and a glossary hover function was used to provide a definition for users.

#### Expert Review of Information for Aboriginal and Torres Strait Islander People

The preliminary site structure for the *Cracks in the Ice* toolkit included a webpage containing a directory of key support services (by state and territory) specific to Aboriginal and Torres Strait Islanders. Key support services and their relevant contact information (eg, phone number, website, email, and postal address) were collated during the scoping exercise described previously. To ensure the information was presented in a culturally appropriate manner, an Indigenous creative agency was engaged to review the content and language and to create the design elements used on the page. Recommendations made included simplifying the language used throughout the webpage, including more personal language (eg, “you” and “your”), reordering of content to reflect that much of the key toolkit information (eg, what is ice, its effects, and how to stay safe) was also relevant to Indigenous communities, and use of landscape imagery (eg, desert or beach) to represent the different states and territories across Australia.

### Phase 3: Beta Testing of the Web-Based Toolkit

#### Design and Procedure

Following Phases 1 and 2, a beta version of the *Cracks in the Ice Community Toolkit* website was developed. Beta testing was conducted among community members and the EAG to ensure the Web-based toolkit was appealing, easy to understand, and engaging, before being launched to the general public. Individuals who participated in the initial end user consultation survey, and who agreed to being contacted in the future, were sent an email invitation to participate in the second phase of consultation. Participants were also recruited via social media channels (Facebook and Twitter) and the research team’s personal and professional networks. Recruitment and beta testing took place over a 5-week period during August and September 2016. Respondents were required to be over the age of 16 years and to be living in Australia to be eligible to take part, and all participants were required to provide informed consent. All participants were asked to view a beta version of the *Cracks in the Ice* toolkit via a preview site and to complete a Web-based survey (of approximately 60 min) to provide in-depth feedback about the website. Members of the EAG (n=10) were also invited to participate in in-house beta testing via the Web-based survey. Approval was obtained from the UNSW HREC (HC15732). A full copy of the questionnaire is available on request.

#### Measures

Respondents were asked to provide closed and open-ended feedback about the ease of navigation, whether the content was easy to understand and informative, how much they liked or disliked the images and infographics used on the website, their likelihood of engaging with specific toolkit features (eg, a quiz or bookmarking functionality to save resources), and overall impressions.

## Results

### Phase 1: End User Consultation

#### Participants

A total of 451 participants provided consent and completed the Web-based survey. Participants ranged in age from 16 to 71 years (mean 27.97 [SD 13.58] years), and 54.8% (246/451) were female. The vast majority of participants (410/451, 91.5%) were born in Australia, with approximately one-third (151/451, 34.0%) residing in New South Wales (NSW), a quarter in Victoria (100/451, 22.5%), and 17.6% (78/451) in Queensland at the time of the survey. Most respondents were employed (279/451, 61.6%), although 10.5% (45/451) were unemployed and more than one-quarter (125/451, 27.9%) were students. The sample included a variety of different groups, including young persons aged 16 to 24 years (243/451, 53.9%), parents (138/451, 30.6%), health professionals (23/451, 5.1%), and teachers (9/451, 2.0%). A total of 4.9% (22/451) of the sample identified as Aboriginal or Torres Strait Islander.

#### Patterns of Methamphetamine Use

Of the 451 survey participants, 37.8% (155/451) had used some form of methamphetamine in their lifetime and 30.4% (137/451) had used crystal methamphetamine (ice). Of those who had used crystal methamphetamine, 32.8% (44/134) reported using weekly or more, 7.5% (10/134) used monthly, 18.7% (25/134) used more than once a year but less than monthly, 10.4% (14/130) had used once in the past year, and 30.6% (41/134) had not used in the past year. The length of crystal methamphetamine use ranged from “less than 1 month” (8/130, 6.2%) to “11 years or more” (13/130, 10.0%), with 68.4% (89/130) using for 1 year or more. The most common route of administration was smoking (125/137, 91.2%); however, nearly one-quarter (32/137, 23.4%) had also injected crystal methamphetamine in the past. The majority of users (95/137, 69.3%) reported using other drugs alongside crystal methamphetamine, most commonly cannabis (70/137, 51.1%), alcohol (58/137, 49.6%), and tobacco (59/137, 43.1%).

#### Reasons for Using Crystal Methamphetamine

Table 1 summarizes the reasons given for using crystal methamphetamine among those who reported crystal methamphetamine use. The most commonly endorsed reasons among lifetime and weekly users were the following: “I like the feeling of being high” (lifetime: 75/137, 54.7%; weekly: 26/44, 59%) and “to party or socialize” (lifetime: 51/137, 37.2%; weekly: 25/44, 57%).

#### Help-Seeking Preferences

When asked where they would be comfortable seeking help if they needed help for their ice use, the most common responses were a “friend or family member” (52/137, 38.0%), counselor or psychologist (42/137, 30.7%), and general practitioner (39/137, 28.5%). Moreover, 21.9% (30/137) of crystal methamphetamine users indicated that they would be comfortable seeking help online; however, one-quarter (34/137, 24.8%) said that they would not feel comfortable asking for help at all. Additionally, 51.2% (197/385) of all participants indicated that they did not feel that effective treatments were available for people seeking help related to the use of ice.

#### Social Harms Resulting From Someone Else’s Use of Crystal Methamphetamine

Harms associated with someone else’s crystal methamphetamine use were prevalent among our sample, with 60.5% (273/451) reporting at least one social harm (mean 2.37 [SD 2.75]). The most commonly reported harms were as follows: having serious arguments or quarrels (162/451, 35.9%), friendship breakdown (149/451, 33.0%), and being insulted or humiliated (138/451, 30.6%; see Table 2).

**Table 1:** Reasons for using crystal methamphetamine among ice users.

Reason for use	Ever used (N=137), n (%)^a^	Used weekly or more (N=44), n (%)^a^
I like the feeling of being high	75 (54.7)	26 (59)
To escape reality	53 (38.7)	25 (57)
To party or socialize	51 (37.2)	13 (30)
To avoid dealing with issues and thoughts	46 (33.6)	19 (43)
To feel confident	45 (32.8)	16 (36)
To think more clearly	38 (27.7)	17 (39)
All my friends use it	28 (20.4)	7 (16)
I used it a bit and now I cannot stop	22 (16.1)	13 (30)
To help me focus at work	12 (8.8)	7 (16)

^a^Participants could endorse multiple reasons.

**Table 2:** Social harms associated with someone else’s use of crystal methamphetamine (N=451).

Harm	n (%)
Ever had serious arguments or quarrels as a result of someone using ice	162 (35.9)
Ever had friendships break up as a result of someone using ice	149 (33.0)
Ever been insulted or humiliated by someone using ice	138 (30.6)
Ever had family problems or marriage difficulties because of someone using ice	130 (28.8)
Ever been a passenger with a driver who has been using ice	127 (28.2)
Ever had financial trouble as a result of someone using ice	103 (22.8)
Ever been disturbed by loud parties or the behavior of someone using ice	99 (22.0)
Ever been pushed, hit, or assaulted by someone using ice	86 (19.1)
Ever had your property vandalized by someone using ice	75 (16.6)

**Table 3:** Reasons for visiting a website about crystal methamphetamine (N=451).

Reason	n (%)^a^
To get information for myself	185 (41.0)
To find out how to get help for a friend or a family member	136 (30.2)
I have heard the term “ice epidemic” and want more information	121 (26.8)
To get information for a friend or a family member	123 (27.3)
To find out how to get help for myself	44 (9.8)
I am a health professional and want more information to help a client	26 (5.8)
I am interested in trying ice in the future and want to know more information before I do it	12 (2.7)
I am a teacher and am looking for educational resources to use in class	3 (0.7)

^a^Participants could endorse multiple reasons.

#### Information Needs About Crystal Methamphetamine

The mostly commonly endorsed reasons for visiting a website about crystal methamphetamine were to “seek information for myself” (185/451, 41.0%) and “to find out how to get help for a friend or a family member” (136/451, 30.2%; see Table 3). In terms of preferences for topic areas, respondents were most interested in obtaining information about the “effects of ice” (199/451, 44.1%), “why do people use ice?” (134/451, 29.7%), and “how many people use ice?” (125/451, 27.7%). When asked to provide open-ended feedback, participants indicated they wanted additional information about a range of topics, including how to help a family member using ice, how to protect yourself from aggressive behavior, the long-term effects of ice use, ice and the law, and warning signs of dependence.

### Phase 2: Content and Web Development

Content development was informed by the Web-based survey with end users and the EAG, literature searching and scoping of evidence, and consultation with external collaborators. The beta version of the toolkit was structured to provide information for the general Australian community across 3 key areas: ice itself (“What is ice?”), its physical and mental health effects (“What are the effects of ice?”), and where and how to access support or treatment for issues related to ice (“Staying Safe”). The toolkit also included user-specific information and resources for community groups and organizations, families and friends of someone using crystal methamphetamine, health professionals and emergency services workers, and schools (parents, teachers, and students). In addition to desktop computers, the toolkit was also optimized for mobile phones, laptops, and tablet devices. A hard-copy booklet was also developed as a companion resource to promote the Web-based toolkit and to provide community groups (eg, support groups, local drug action teams), health professionals, and individuals with tangible *Cracks in the Ice* resources and information about crystal methamphetamine. The decision to develop and distribute hard-copy booklets was made in consultation with the EAG and is consistent with previous dissemination activities conducted by the research team.

### Phase 3: Beta Testing of the Web-Based Toolkit

#### Participants

A total of 41 participants completed the Web-based survey. The mean age of participants was 34.4 years (SD 15.0) and 49% (20/41) of the sample were female. Nearly half of the sample (19/41, 46%) resided in NSW and almost one-quarter in Victoria (9/41, 22%), with 61% (25/41) of all participants living in metropolitan areas at the time of the survey and 39% (16/41) in regional or rural areas. Over two-thirds of the sample (28/41, 68%) indicated that they knew someone who uses ice, with 39% (16/41) identifying themselves as a friend of an ice user and 20% (8/41) as a family member of someone who uses ice. The sample consisted of individuals representing a variety of different groups, including parents (16/41, 39%), students (15/41, 37%), health professionals (7/41, 17%), and teachers (2/41, 5%).

Participants viewed the beta site across a variety of Web browsers (Chrome [16/41, 39%], Safari [11/41, 27%], Firefox [6/41, 15%], and Internet Explorer [5/41, 12%]) and multiple devices (mobile phone [18/41, 47%], laptop [10/41, 26%], desktop computer [5/41, 13%], and tablets [5/41, 13%]).

#### Feedback

Overall, feedback on the beta version of the *Cracks in the Ice Community Toolkit* was positive in terms of content, readability, layout, and aesthetic quality of the site. All community participants (41/41, 100%) rated the content as easy to understand and informative, and the vast majority (38/41, 93%) rated the site’s navigation as “good” or “very good.” Example qualitative feedback is provided in Multimedia Appendix 2. The EAG was also positive in its feedback about the Web-based toolkit, with the main areas for improvement relating to the need to improve the ease of navigation and layout of information on some content pages (see Multimedia Appendix 2 for example feedback). Three quarters of participants (28/37, 75%) indicated that they liked the toolkit’s mission statement “Trusted, evidence-based information about crystal methamphetamine,” and more than half (20/37, 54%) indicated that an evidence base was most important when looking for information about crystal methamphetamine.

#### Summary of Modifications

**Figure 2:**
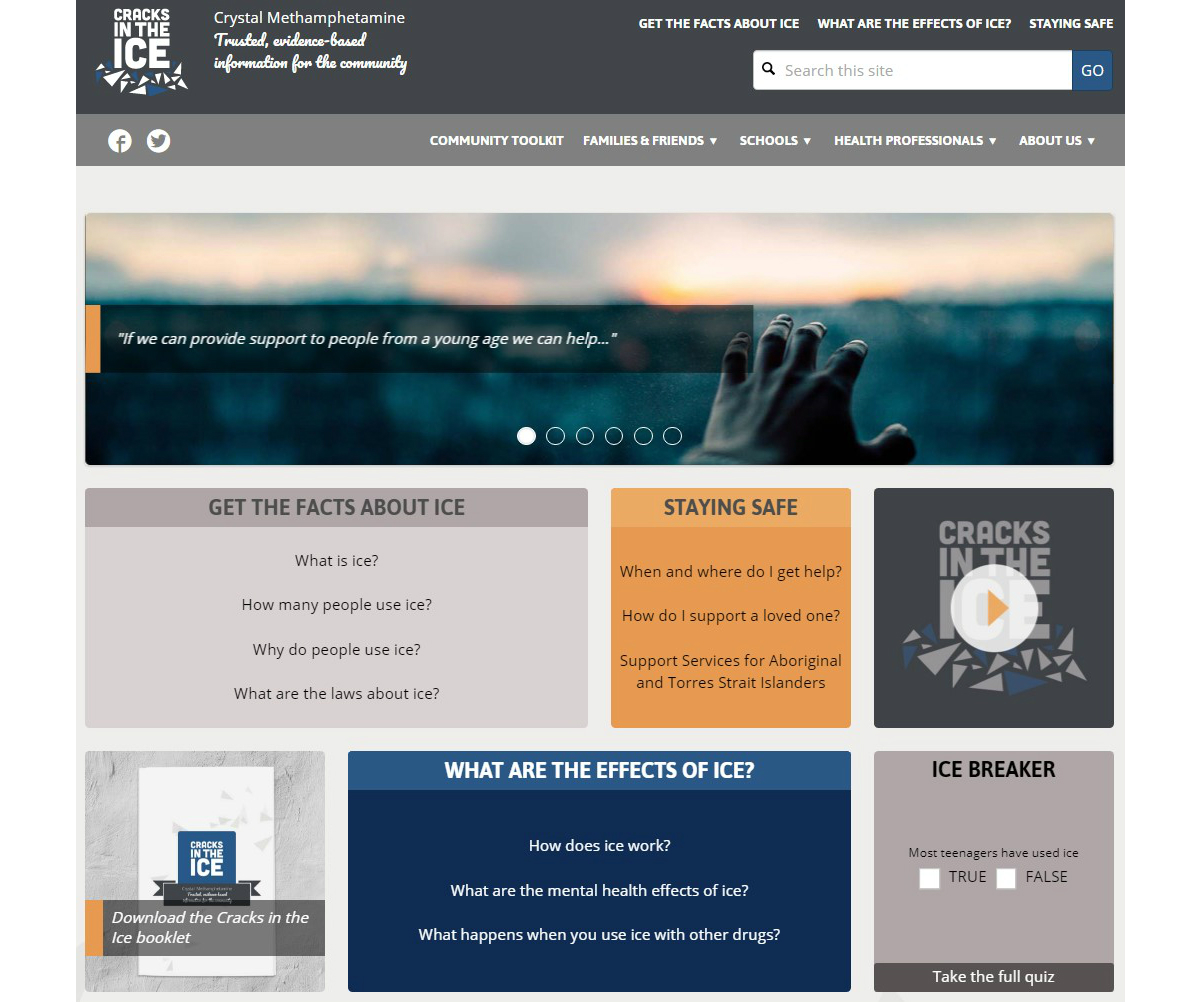
The Cracks in the Ice Community Toolkit home page (at time of launch).

**Figure 3:**
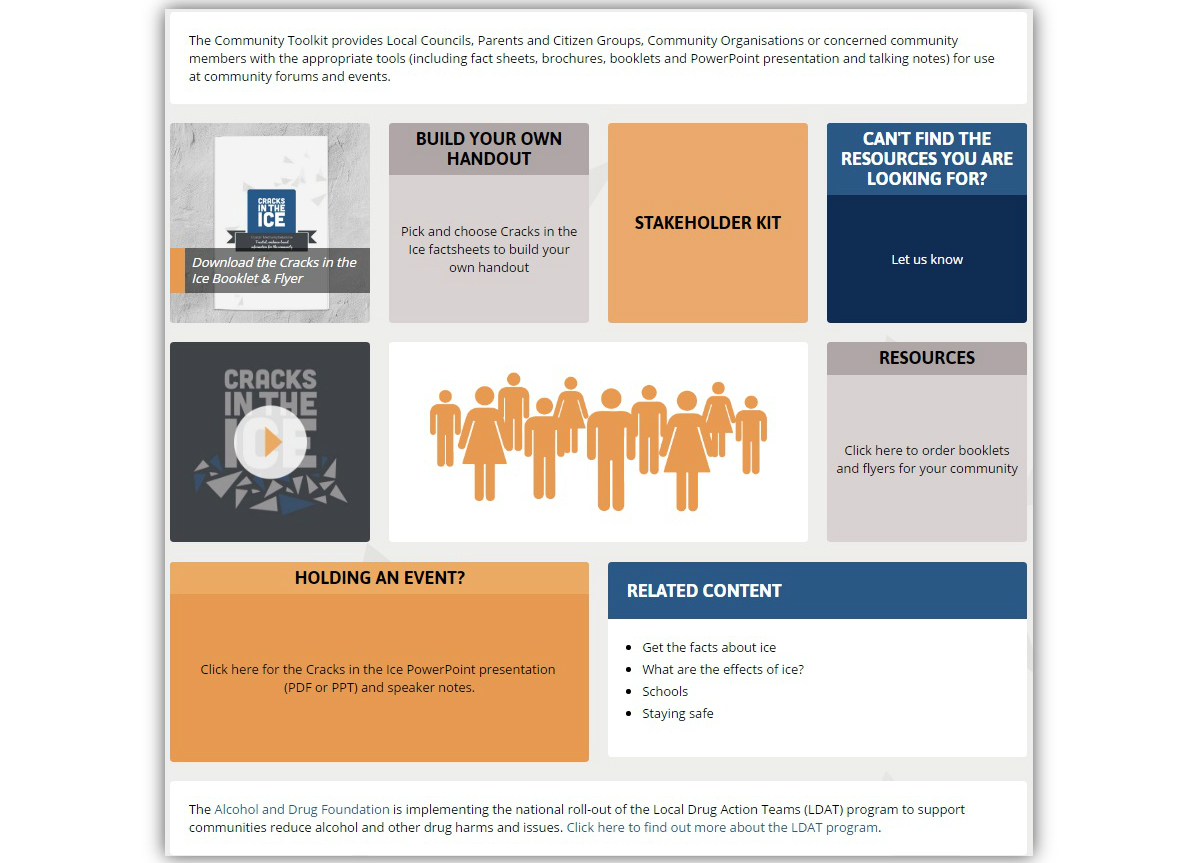
Screenshot of the Community Toolkit page (at time of launch).

**Figure 4:**
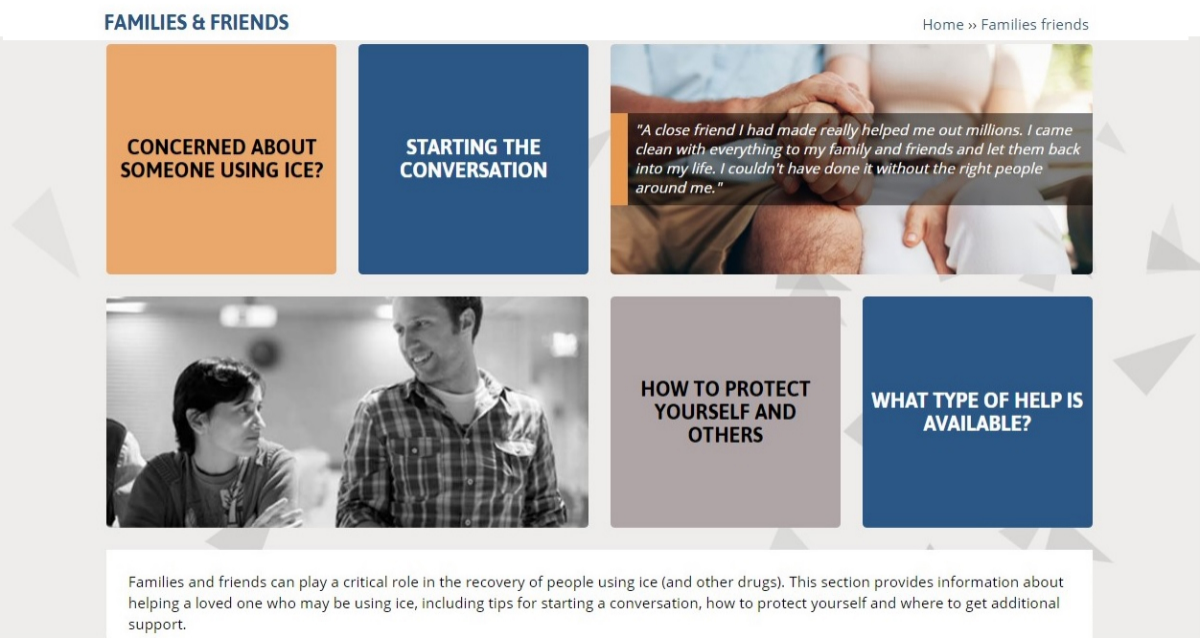
Screenshot of the Families & Friends page (at time of launch).

The most commonly identified areas for improvement were related to increasing the level of engagement and personal connection with the site, improving ease of navigation, and balancing the “low prevalence of use, yet high impact” message. To address these concerns, a number of modifications were made to the Web-based toolkit. To achieve greater user engagement and personal connection, additional images and infographics were included and quotes based on personal experiences related to methamphetamine use were embedded on all key pages of the website. In addition, content was modified on the “How many people use ice?” webpage to reflect the difficulties in measuring methamphetamine use in the general population and to convey the message that although rates of use might be low, the impact of crystal methamphetamine use on individuals, families, and communities can be substantial. The project team also worked in consultation with the Web developers to improve the layout and ease of navigation, for example, including “Related Content” links on each page and Show and Hide functionality to collapse large amounts of text into smaller sections. Tables 4 and 5 summarize the key content included on the final *Cracks in the Ice* website at the time of launch to the general public, and Figures 2-4 provide screenshots of key toolkit webpages.

**Table 4:** Overview of key content on the *Cracks in the Ice* website.

Overarching topic		Content
**Get the Facts about Ice**		
	What is ice?	Definition of methamphetamine and its different forms (“ice,” base, and speed) Information about the appearance, route of administration, purity, and street names for methamphetamines
	How many people use ice?	Prevalence of methamphetamine use and related harms in Australia Trends in methamphetamine use and harms in Australia
	Why do people use ice?	Commonly reported reasons for using crystal methamphetamine Reasons young people might use drugs, such as crystal methamphetamine Tips for being assertive and drug refusal skills
	What are the laws about ice?	Summary of the law for methamphetamine (including crystal methamphetamine)-related offences in Australia and by state and territory
What are the effects of Ice		How methamphetamine affects the brain and body The short- and long-term mental health effects of methamphetamine use Using “ice” with other drugs, including reasons for polydrug use and the effects of using ice with stimulants, depressants, and with medications
Staying Safe		When and where to get help, including a self-assessment tool to provide feedback about one’s use of crystal methamphetamine How to support a loved one who is using methamphetamine Support services for Aboriginal and Torres Strait Islanders

**Table 5:** Overview of user-specific content on the *Cracks in the Ice* website.

User group	Description
Community toolkit	The community toolkit provides local councils, parents and citizen groups, community organizations, or concerned community members with the appropriate tools (including factsheets, brochures, booklets, and PowerPoint presentation and talking notes) for use at community forums and events
Families and friends	This section provides information about helping a loved one who may be using ice, including tips for starting a conversation, how to protect yourself, and where to get additional support
Health professionals	Factsheets, guidelines, and Web-based resources for professionals working across a range of sectors, including general practitioners, frontline workers in hospital settings and emergency departments, frontline workers in alcohol and other drug settings, mental health practitioners (eg, psychologists, social workers, and counselors), paramedics, and police services
Schools	Access to information and resources for parents, teachers, and students, including webinars, factsheets, and interactive games

### Usage Statistics

The *Cracks in the Ice Community Toolkit* was officially launched on April 3, 2017. In the 3 months immediately post launch, the toolkit received a total of 9138 visitors. Excluding the homepage, the most frequently visited toolkit pages were the following: How many people use ice?, the Community Toolkit, and What is ice?. Since launch, a total of 25,480 hard-copy *Cracks in the Ice* resources (14,040 booklets and 11,440 flyers) have been distributed to individuals, organizations, and communities across Australia. Of these resources, 60.93% (15,525/25,480) were distributed to relevant organizations and subscribers on the *Cracks in the Ice* mailing list, and 39.07% (9955/25,480) were ordered directly by individuals and organizations via the *Cracks in the Ice* website.

A full evaluation of engagement with the website is planned to determine who is visiting the website, the usefulness of the Web-based toolkit among visitors, and to better understand how people respond to the information provided in the toolkit. These data will be used to better tailor and target the information and resources on the *Cracks in the Ice Community Toolkit.*

## Discussion

### Summary of Findings

This paper described the codevelopment process of *Cracks in the Ice,* a Web-based toolkit designed to provide evidence-based information and resources about crystal methamphetamine for the Australian community. The codevelopment process was broad-reaching and iterative, involving more than 450 community members across the country and expert collaborators and researchers over an 18-month period. The initial Web-based survey conducted with end users provided important insights into the information needs of the Australian community in relation to crystal methamphetamine and validated the preliminary site structure. Results indicated that community members were seeking information about crystal methamphetamine, particularly in relation to its effects and reasons for use, and were likely to visit a Web-based toolkit to search for information for themselves and to help a friend or family member. Social harms related to the use of crystal methamphetamine were considerable in our sample, with nearly one-third of the respondents reporting 1 or more harm related to someone’s use of the drug. Overall, 63.6% (287/451) of the Web-based survey sample reported either having ever used crystal methamphetamine or experiencing at least one social harm from someone else’s use of ice Thus, the majority of the sample had lived experience of the impacts of crystal methamphetamine use, either through their own use or that of someone else.

Consistent with previous research [27,28], participants in this study indicated that their preferred source of help would be from friends and family members. One in 4 ice users from our Web-based scoping survey said that they would not feel comfortable seeking any help for their ice use and only 21.9% (30/137) said they would be comfortable seeking help online. Additionally, just over half of the whole sample (197/385, 51.2%) said they did not feel effective treatments were available for methamphetamine use. Stigma and the belief that no effective help is available are 2 of the most commonly stated barriers to help-seeking for substance use and mental health problems [29-31]. Internet-based interventions do hold promise to overcome these barriers to care [32,33], particularly if information can be presented in a nonjudgmental and stigmatizing manner [34]. Further research is needed to clarify the reasons why some individuals do not feel comfortable seeking help online for their methamphetamine use (eg, privacy or stigma) and to understand how these concerns can be addressed in online treatment delivery. Importantly, the *Cracks in the Ice Community Toolkit* was developed to provide evidence-based information about ice, including information about when and where to get help, and what types of treatment are available, but not as a treatment intervention. Results from the beta testing survey indicated that the Web-based toolkit was well received by end users as a source of information about ice.

Overall, the beta-testing results were overwhelmingly positive and served to both reinforce the development work conducted thus far and further refine the toolkit to ensure it was as relevant, acceptable, and appealing as possible for the target audience. Three key areas for improvement emerged from the beta-testing process. First, end users indicated that they wanted an increased level of engagement and personal connection with the site. In response to this, additional images and infographics were included across the website. Specifically, photos were paired with real quotes from individuals about their experiences related to methamphetamine and prominently displayed on all key pages across the *Cracks in the Ice* site. For example, the homepage was modified to include a rotating banner of different pairs of images and quotes, for example, “If we can provide support to people form a young age we can help.” Second, beta-testing data indicated that there was a clear need to improve the ease of navigation on the website to ensure that site users could easily locate the information they were seeking. To improve navigation, the project team worked in consultation with the Web developers to modify the layout across several webpages, for example, including drop-down menus on the homepage, adding “Related Content” links on each page to allow users to easily navigate to similar content, and using the Show and Hide functionality to collapse large amounts of text into smaller sections. Third, beta testing revealed that there was a need to better covey a “low prevalence of use, yet high impact” message. To address this feedback, text was modified on the How many people use ice? webpage to reflect the difficulties in measuring methamphetamine use in the general population, and additional sources of data on harms were included. In addition, an infographic was developed to convey the message that although rates of use might be low, the impact of crystal methamphetamine use on individuals, families, and communities is substantial.

### Community Response and Future Directions

Importantly, beta-testing data confirmed that end users valued the toolkit’s emphasis on providing evidence-based information, with more than half of participants indicating that an evidence base was the most important aspect when looking for information about crystal methamphetamine. Given the considerable focus on ice use in Australia in recent times and the potential for misinformation about crystal methamphetamine to be disseminated via the media and the internet, a key strength of the *Cracks in the Ice Community Toolkit* is the ease of access to evidence-based information and resources. To ensure that the information and resources remain evidence-based and up-to-date, important next steps for the maintenance of the website will be to review the academic literature and other relevant sources at regular intervals in the future and to update the website accordingly. Since its launch, community response has been positive, with steady increases in visitors to the *Cracks in the Ice* website and regular requests to receive hard-copy booklets and flyers. The dissemination and early demand for hard-copy resources is another strength of *Cracks in the Ice*, with booklets ordered by, and distributed to, a range of groups across the country, including drug and alcohol services, community centers, and support groups. Although developed as a companion resource to the Web-based toolkit, dissemination of hard-copy resources may be important for providing support to people who are less familiar with Web-based resources, or those without easy access to computers or the internet. Ultimately, the final *Cracks in the Ice Community Toolkit* provides Australians, including community groups, families and friends of someone using ice, health professionals and emergency service workers, and individuals affected by crystal methamphetamine, with the much-needed evidence-based and up-to-date information and support, in one easily accessible Web-based toolkit.

### Limitations

The findings presented in this paper should be considered in light of some limitations. First, the sample recruited for the initial end user consultation was not representative of the general Australian population, and prevalence of methamphetamine use (155/451, 37.8%), including ice, was significantly higher than the population rates [3]. Although we did not primarily target methamphetamine users in our recruitment advertisements, it is likely that our strategy attracted individuals with a particular concern about methamphetamine use, that is, individuals who use ice and their friends or family members. The Web-based consultation survey was also restricted to individuals who use the internet, and in particular, Facebook. Nonetheless, the survey methodology was successful in identifying key knowledge gaps and information needs in the community, as well as design and aesthetic preferences for the website, thereby directly informing the development of the Web-based toolkit. It should also be noted that the consultation survey was completed by only a small number of teachers and health professionals, 2 target audiences of the toolkit. Importantly, the content housed on *Cracks in the Ice* for these 2 groups primarily draws on existing resources that were previously developed in consultation with end users (eg, the *Positive Choices* website, which was developed with input from teachers, and guidelines developed by expert health professionals). Nonetheless, there is opportunity for future development work for the toolkit to involve further consultation with teachers and health professionals. Similarly, beta testing was conducted among a relatively small sample, via convenience sampling, and may not be representative of the general population. Fortunately, the *Cracks in the Ice Community Toolkit* contains feedback loops and opportunities for website users to submit questions and provide feedback, ensuring the continuing involvement of additional community members as the site is maintained into the future.

### Conclusions

This recently developed *Cracks in the Ice Community Toolkit* involved in-depth community consultation and brings together the latest evidence and resources pertaining to crystal methamphetamine in Australia. Ultimately, the toolkit provides the Australian community with a central access point for trusted, evidence-based, and up-to-date information about crystal methamphetamine. Important next steps are to monitor engagement and use of the website, to embed the most recent evidence into the Web-based toolkit to ensure the currency of information, and to remain responsive to emerging information needs about crystal methamphetamine among the community.

## Appendix

Multimedia Appendix 1 Adapted version of the National Health and Medical Research Council body of evidence matrix.

Multimedia Appendix 2 Example feedback from end users and the Expert Advisory Group about the beta-version of Cracks in the Ice.

## Abbreviations

EAG: Expert Advisory Group
HREC: Human Research Ethics Committee
NSW: New South Wales
UNSW: University of New South Wales
