# Supplementary material for: A Web-Based Toolkit to Provide Evidence-Based Resources About Crystal Methamphetamine for the Australian Community: Collaborative Development of Cracks in the Ice
Source: JMIR Ment Health. 2018 Mar 20;5(1):e21. doi: 10.2196/mental.8891 (PMC5883077; doi:10.2196/mental.8891)
Supplement: Multimedia Appendix 1 [file mental_v5i1e21_app1.pdf]

Multimedia Appendix 1: Adapted version of the NHMRC Body of Evidence Matrix[1]

**Criteria**	**A**	**B**	**C**	**D**
	**Excellent**	**Good**	**Satisfactory**	**Poor**
**Evidence base:** Was the resource developed on the basis of evidence?	Formally evaluated and findings published.	No formal evaluations; Developed on the basis of published findings OR some testing among end-users has been conducted.	No reference to formal evidence or testing; developed with formal input from experts.	Developed on basis of personal opinion only/unknown.
**Impact and utility[2]:** Range and importance of issues covered	**Very large**: covers a range of relevant issues comprehensivel y.	**Substantial**: covers more than one relevant issue.	**Moderate**: covers a single issue of high importance.	**Slight or restricted**: brief resource, covers a single issue of lesser importance.
**Generalisability:** Relevance of the resource to the community and/or target groups for the toolkit	Relevant to one or more of the toolkit's target groups.			Not relevant for any of the toolkit's target groups.
**Applicability:** How applicable is the resource to an Australian context?	Directly applicable to Australian context.	Applicable to Australian context with some caveats.		Not applicable to Australian healthcare context.

[1] The original NHMRC Body of Evidence Matrix included an additional criterion 'Consistency' (whether the findings are consistent across the included

studies). This component was omitted from the present study as single resources were evaluated one at a time.

[2] This criterion was labelled ‘Clinical Impact’ in the original NHMRC Body of Evidence Matrix. This component was modified to ‘Impact and utility’ to reflect that the target audience of the online toolkit is largely non-clinical i.e. the Australian community.

External resources were assessed according to the criteria outlined above and using the following steps:

- Prior to the formal assessment process, three resources were rated by all project team members and ratings were discussed to ensure the validity of the scale for this purpose.
- Resources needed to score a C (a satisfactory rating) or above on all four components of the scale, except in exceptional circumstances (e.g. a rating of D for impact and utility, with ratings of A or B across the other three domains).

Each resource was reviewed by one member of the team. If the resource did not receive a score that warranted inclusion, the resource was discussed at a team meeting, and if consensus could not be reached, external expert opinion was sought. Only one resource was deemed ineligible for inclusion on the toolkit and was excluded.
