# Supplementary material for: A Web-Based Toolkit to Provide Evidence-Based Resources About Crystal Methamphetamine for the Australian Community: Collaborative Development of Cracks in the Ice
Source: JMIR Ment Health. 2018 Mar 20;5(1):e21. doi: 10.2196/mental.8891 (PMC5883077; doi:10.2196/mental.8891)
Supplement: Multimedia Appendix 2 [file mental_v5i1e21_app2.pdf]

Multimedia Appendix 2: Example feedback from end-users and the Expert Advisory Group about the beta-version of Cracks in the Ice.

Source	Example feedback
Community	*"It's informative and interactive and easy to find information."*
Community	*"It's easy to navigate for people who could be distressed and rushing to help a friend or family member."*
Community	*"I like the images as they are relevant and portray that the website is for anyone to use. I also like that they aren't stereotypical."*
Community	*"I like 'Starting the Conversation' as this is one of the hardest parts of dealing with an ice user. It is well set out and understandable."*
Community	*"The variety of different sources people can access for help is great."*
Expert	*"It is very static. Perhaps as part of future development we could add some movement eg, rotating images."*
Expert	*"Friendly, inclusive, simple to navigate."*
Expert	*"I think a glossary of unfamiliar terms would be useful."*
